# Studying trait-playfulness, time spent with physical activity, and athletic identity among self-ascribed athletes and non-athletes

**DOI:** 10.3389/fspor.2025.1669367

**Published:** 2025-10-14

**Authors:** Kay Brauer, Johanna E. Donhauser, René T. Proyer

**Affiliations:** Department of Psychology, Martin Luther University Halle-Wittenberg, Halle (Saale), Germany

**Keywords:** playfulness, athletes, sports, athletic identity, physical activity

## Abstract

Adult playfulness is an individual difference variable that describes how people (re)frame situations in a way that they are experienced as entertaining, and/or intellectually stimulating, and/or personally interesting. Playfulness relates to indicators of mental and physical health, but its role among athletes is yet understudied. In our study, we provide initial findings on playfulness with regard to self-reported athleticism by (a) comparing expressions in four facets of playfulness (Other-directed, Lighthearted, Intellectual, and Whimsical types) between athletes (*n* = 205) and non-athletes (*n* = 209), and (b) testing associations with subjective impressions of athletic identity (i.e., the degree of one's identification of being an athlete) and reports of time spent with physical activity. Our findings showed that (a) there is measurement invariance of playfulness among the groups, (b) athletes yield higher expressions of Lighthearted playfulness (*g* = 0.31), and among athletes, playfulness goes along with more time spent participating in physical activity whereas it was unrelated to athletic identity; among non-athletes, Other-directed playfulness related to perceiving oneself as being athletic. We discuss our findings regarding implications for leisure and performance-related outcomes and in line with the literature noting the important role of playfulness in sports.

## 1 Introduction

“*I’m just a hockey player. I want to play every day.*”—Martin Brodeur (former professional ice hockey goaltender)

The benefits of participating in physical activity (PA) for physical and mental health are well studied (1, 2), but engagement in PA is declining globally (3). As illustrated in Brodeur's quote, many athletes view and speak of their physical activity^1^ as “play,” and there is increasing interest in understanding what contributes to athletes and non-athletes taking part in PA from the perspective of play and playfulness [e.g., (4–6)]. While earlier research examined the role of playful approaches for designing PA programs, creating environments that enable immersion, and using gamification, our study extends the knowledge of the field by studying playfulness as a personality trait. Therefore, we compared expressions in four facets of playfulness among self-ascribed athletes and non-athletes and examined its relations with their athletic identity and time spent engaged in PA.

### 1.1 Adult playfulness

Playfulness is a personality trait that describes individual differences in (re)framing situations in such a way that they are experienced as personally interesting, and/or intellectually stimulating, and/or entertaining (7). Playfulness, as a trait, is relatively stable across situations and time and describes inclinations to engage in play, the behavior. The OLIW-model of adult playfulness (7) differentiates between four facets of playfulness, including: ***O***ther-directed (i.e., enjoying playing with others, using playful approaches in social relationships); ***L***ighthearted (i.e., liking improvising, seeing life as a game rather than a battlefield); ***I***ntellectual (i.e., taking pleasure in playing with ideas and thoughts, preferring complexity over simplicity); and ***W***himsical (i.e., enjoying unusual hobbies, finding amusement in grotesque situations. The facet approach allows distinguishing between how people engage in playful behaviors, as people can show different profiles in expressions of the four facets. For example, a person may demonstrate Other-directed playfulness, moderate levels of playfulness, but lower degrees of Intellectual and Whimsical playfulness. The facetted nature of this approach to playfulness allows to investigate and describe individual differences in playfulness in a fine-grained manner. The OLIW model also covers intellectual components and less fun-oriented aspects that are often neglected in the literature, which allow to learn more about the role of playfulness in contexts that require a serious approach beyond mere fun and entertainment [see (8), for an overview]. Numerous studies have shown that the facets show differential relations with external outcomes, such as relationship satisfaction in couples, responses to traumatic experiences during war, dealing with stress, creativity, and well-being, to name but a few [e.g., (9–13)]. Since engaging in sports and physical activity is often challenging and requires serious approaches, we chose the OLIW model to learn more about the role of playfulness among athletes’ and non-athletes’ engagement in being active.

### 1.2 Playfulness and physical activity

Children's playfulness relates to engaging in greater activity and higher physical fitness, practicing their motor skills (14, 15). Adults engage in PA for different reasons, including to increase one's physical and mental health, or because they enjoy playing with others [e.g., in team sports; see (16)]. Recently, the role of play and playfulness has been identified as an important quality because incorporating playful elements into exercises contributes to subjective and objective performance parameters and flow experience (4–6, 17). This fits well with studies showing that boredom can negatively affect engaging in sports. Velasco and Jorda (18) identified several types of issues that lead to boredom (e.g., monotonous activities; anticipated negative mood; lack of competitiveness and challenges) among athletes and translates to consequences such as reduced time spent in PA. Accordingly, one would expect that being able to reframe such situations in a playful manner—for example, by setting small challenges, sport-related daydreaming, or imagining different outcomes of training sessions and/or competitions—will contribute to overcoming barriers of engaging in PA. Playfulness helps prevent boredom from occurring [e.g., (19)] and may also be helpful in making daily routines in exercises more interesting. A competitive aspect to playfulness could maybe play a role here as well [see e.g., (17, 20)].

Trait-playfulness relates positively to participating in sports, as it contributes to engaging in social play and activities (e.g., team sports) as well as facilitating avenues to learning the ways in which engaging in sports and PA can elicit positive emotions [e.g., (21)]. In line with Verwijmeren et al. (6), who showed that athletes incorporate elements of amusement and challenge into how they approach sports, we expected that playful individuals would spend more time in PA than those low in playfulness because playfulness allows them to approach PA—even including monotonous and repetitive behaviors and movements—in a way that is experienced as interesting, entertaining, or intellectually stimulating. Further, playfulness has been linked to a set of variables that contribute to engaging in PA, such as adaptive approaches to deal with stressors, greater flow experiences which are linked to the ability to (re)frame situations in an interesting and entertaining way, intrinsic- and achievement-related motivation, mastery orientation, and pursuing activity-based leisure [e.g. (13, 22–25)].

Initial data support the notion of playfulness relating to greater PA and fitness: In a study of 252 participants, a global measure of playfulness related positively to the inclination to pursue an active way of life and engage in sports in leisure-time [*r*s = .22 and .24 (25)]. In addition, playfulness related to higher coordination skills according to self-reports. Furthermore, Proyer et al. (26) extended these findings with two studies focusing on the OLIW facets of playfulness as well as objective criteria of fitness. In Study 1 (*N* = 529), the OLIW facets related to higher activity levels and pursuing an active way of life; in Study 2, they invited 67 participants to the lab for interviews and physical exercise tests, including hand-grip strength, a stair-climbing exercise (cardio-respiratory fitness), the sit and reach test (back and leg flexibility), the 1-min sit-to-stand test (lower body muscular strength and endurance), and tests of fine motor functions. The interviews showed that Other-directed and Lighthearted playfulness related to being active in the last seven days (*r*s = .29 and .26), including time spent with moderate and vigorous activity (Other-directed) and walking (Lighthearted), as well as negative relations to time spent sitting (*r*s = −.25 and −.21). Moreover, the facets showed differential relations with the objective indicators of baseline-, activity-, and recovery heart rates, being negatively related to Other-directed playfulness; all facets showed relations to greater hand and forearm strength; and Lighthearted playfulness related to greater lower body strength and endurance. Overall, the available findings and theoretical considerations support the notion that trait-playfulness may contribute to engagement in PA, including its benefits of mental and physical health.

### 1.3 The present study

Despite the increasing interest in play(fulness) in sports, the role of trait-playfulness is hitherto understudied. Specifically, it is unclear whether playful people might engage in more physical activity than less playful people. Accordingly, we addressed this gap in the literature by collecting data from both self-ascribed athletes and non-athletes. In line with the literature [(27); see also (28)], we asked participants to self-identify as athlete or non-athlete. Additionally, we assessed their athletic identity, which describes how strongly and exclusively one identifies with the athlete role (27) and has been found to robustly predict various outcomes such as burnout, substance use, sensitivity to training change, and career transitions (29). This approach allowed us to examine whether there are robust differences in the expressions of facets of playfulness among those who view themselves as athletes or non-athletes, and how the OLIW facets correlate with athletic identity measured on a dimension from none to high athletic identity. We established measurement invariance for playfulness between athletes and non-athletes to clarify whether the scores from the OLIW questionnaire (7) can be compared in a meaningful way between the groups. Finally, we asked participants to report their time spent with PA. Taking earlier findings into account (6, 18, 25, 26), we expected positive associations with the OLIW facets. Our findings will contribute to the knowledge by examining playfulness as a personality trait that might explain differences between athletes and non-athletes and inclinations to engage in physical activity.

## 2 Methods

### 2.1 Sample and procedure

Our sample comprised 414 participants, of which 205 identified as athletes and 209 as non-athletes. The mean ages were 25.6 (*SD* = 7.2; non-athletes) and 27.1 years (*SD* = 7.1; athletes). Of the non-athletes, 80.4% were women, 17.2% men, 1.9% indicated third gender, and 0.5% did not indicate their gender; of the athletes, 61.5% were women, 38.0% were men, and 0.5% did not indicate their gender. As in prior research (30, 31), those who self-identified as athletes reported more time spent with PA at the time of the study (*M* = 8.8 h/week; *SD* = 3.7, 2 to 26 h/week) than non-athletes (*M* = 5.1; *SD* = 3.0; 0 to 15 h/week; Hedges’ *g* = 1.11 95% confidence interval [0.89, 1.30). Overall, the educational status was high, with 31.7% holding a university degree and 54.3% with a high-school diploma qualifying them to attend university, 6.3% had completed vocational training, and the remaining participants held a high-school diploma without qualification to attend university. Power analyses showed that the samples allowed the detection of correlations ≥.17/.20 with 80/90% statistical power (*α* = .05).

We collected all data online (https://soscisurvey.de) between May 2024 and February 2025. We advertised the study online and with leaflets through the authors’ department's website and by contacting sports clubs with amateur and semi-professional members.

### 2.2 Instruments

We assessed playfulness using the OLIW questionnaire (7). The 28-item instrument assesses the four facets, which include: Other-directed (e.g., “I have close friends with whom I can just fool around and be silly”); Lighthearted (e.g., “I do not live from day to day at all; I rather plan ahead long in advance” [reverse); Intellectual (e.g., “I can always think of something to do and I am never bored”); and Whimsical (e.g., “I have an unusual habit or an uncommon hobby”). Participants gave their responses on a 7-point Likert-type rating scale (1 = *strongly disagree*; *7* = *strongly agree*). The internal consistencies were comparable with prior studies [e.g., (10, 32)], with *α* = .70 (Other-directed), .77 (Lighthearted), .59 (Intellectual)^2^, and .81 (Whimsical). There is robust evidence for the reliability and validity [e.g., retest- and inter-rater agreement; self-other agreement; structural validity across data sources; (7, 32, 33)].

We used the 10-item *Athletic Identity Measurement Scale* [AIMS; (27); German version (35)] to assess athletic identity (e.g., “Sport is the most important part of my life”). Participants respond on a 7-point Likert scale (1 = *strongly disagree*; 7 = *strongly agree*). We computed the total score of the AIMS as indicator of athletic identity since Schmid and Seiler (35) noted low discriminant validity of potential subscales. Brewer et al. (27) reported a retest-correlation of .89 across 14 days and a recent meta-analysis of the AIMS provides robust evidence regarding the AIMS’ reliability and validity of the total score (28). In our study, the internal consistency was high (*α* = .94) and comparable to earlier research using the German version (35). Further, Brewer and Cornelius (36) demonstrated measurement invariance between athletes and non-athletes.

### 2.3 Data analysis

We examined the measurement invariance of the OLIW questionnaire between the groups of athletes and non-athletes to establish whether the scale scores can be compared across groups in a meaningful way. We computed the analyses in Mplus 8.8 (37) using the robust maximum-likelihood estimator (38). We computed three models of measurement invariance with increasing constraints, namely: configural invariance (i.e., same number of factors); metric invariance (i.e., setting item-factor loadings equal between groups); and scalar invariance (i.e., assuming equal latent means across groups). We used Chen's (39) combinational guidelines of RMSEA, CFI, and SRMR to evaluate the change of model fit, suggesting that metric invariance should be rejected when *Δ*CFI ≥ .010 and *Δ*RMSEA ≥ 0.015 (or *Δ*SRMR ≥ .030) and scalar invariance should be rejected when *Δ*CFI ≥ .010 and *Δ*RMSEA ≥ 0.015 (or *Δ*SRMR ≥ .010). Further, we computed Hedges’ *g* effect size for comparisons of group means, with coefficients ≥ 0.20/0.50/0.80 indicating small, medium, and large effects.

We computed Pearson correlations to examine the associations between study variables, assuming *r*s ≥ .10, .20, and .30 indicate small, medium, and large effect sizes (40). Finally, we used regression analyses to examine the unique contribution of the playfulness facets beyond age and gender (included in Step 1; method = ENTER) in predicting the outcomes of time spent with PA and athletic identity. Again, we used a standardized effect size measure to evaluate the findings, namely, Cohen's regression effect size *f*^2^ indicating small, medium, and large effects when ≥ 0.02, 0.15, and 0.35.

## 3 Results

### 3.1 Preliminary analyses

Table 1 provides the descriptive statistics. Overall, the total sample showed similar expressions to those found in other German-speaking samples [e.g., (38)]. For athletic identity, the expressions fit well with the range reported in a recent meta-analysis, with our participants who identified as athletes yielding scores that are reported as typical for athletes on the intermediate level [i.e., identify as non-elite, in extensive training; (28)] whereas the scores of the self-ascribed non-athletes align with those reported for non-athletes (41). As expected, athletes reported markedly higher athletic identity than non-athletes [*g* = 2.20, 95% confidence interval (1.95, 2.44)].

**Table 1:** Means and standard deviations of playfulness and athletic identity.

Playfulness	Total (*N* = 414)		Non-athletes (*n* = 209)		Athletes (*n* = 205)		Hedges’ *g*
	*M*	*SD*	*M*	*SD*	*M*	*SD*	
Other-directed	5.08	0.90	5.11	0.85	5.05	0.95	−0.07 [−0.26, 0.13]
Lighthearted	3.95	1.03	3.78	1.05	4.10	1.00	0.31 [0.12, 0.51]
Intellectual	4.09	0.82	4.05	0.84	4.13	0.79	0.10 [−0.10, 0.29]
Whimsical	3.93	1.06	3.84	1.01	4.01	1.11	0.16 [−0.03, 0.35]
Athletic Identity	3.39	1.59	2.23	0.90	4.58	1.22	2.20 [1.95, 2.44]

Bootstrapped 95% confidence intervals from 5,000 random samples in brackets.

### 3.2 Comparing athletes’ and non-athletes’ playfulness

Our measurement invariance analyses showed no evidence for rejecting metric and scalar invariance (all *Δ*RSMEAs ≤ 0.001, *Δ*SRMRs ≤ 0.004, and *Δ*CFIs ≤ 0.008). Hence, the manifest scores of the OLIW questionnaire can be compared between our athletes and non-athletes.

We compared the athletes and non-athletes concerning their expressions in the facets of playfulness and found one notable group difference, namely the athletes scored higher in Lighthearted playfulness, with a small effect size [*g* = 0.31 (0.12, 0.51); Table 1]. We found no robust differences for the remaining facets.

### 3.3 Playfulness and athletic identity

Table 2 gives the correlations between the OLIW facets and the AIMS. While they were independent when considering the total sample and athletes, we found minor associations between athletic identity and Other-directed and Lighthearted playfulness, with coefficients of *r* = .17 and .15 (*p*s = .012 and .027) in non-athletes. Overall, the OLIW facets explained 5.8% of variance in athletic identity (*F*_4,202_ = 3.15, *p* = .015), a small effect size (*f*^2^ = 0.06) beyond age and gender (*R*^2^ = .01; *f* = 0.01) in non-athletes. An inspection of the predictors showed that Other-directed entered the model with a small effect size (*β* = .20, *p* = .016, *f*^2^ = 0.03). On the contrary, playfulness was unrelated to athletic identity among self-ascribed athletes (*r*s ≤ |.09|, *p* ≥ .200), explaining 2.2% shared variance (*F*_4,198_ = 1.16, *p* = .331) beyond age and gender (*R*^2^ = .02; *f*^2^ = 0.02)^3^.

**Table 2:** Partial correlations between athletic identity and facets of playfulness.

Group	Other-directed	Lighthearted	Intellectual	Whimsical
Total	.06 [−.05, .16]	.08 [−.02, .17]	.05 [−.05, .14]	.05 [−.05, .15]
Non-athletes	.17* [.04, .31]	.15* [.02, .29]	.06 [−.07, .18]	−.01 [−.15, .12]
Athletes	.07 [−.08, .22]	−.09 [−.24, .05]	.01 [−.13, .15]	.04 [−.11, .18]

* *p* < .05. Two-tailed. Bootstrapped 95% confidence intervals from 5,000 random samples in brackets. Controlled for age and gender.

### 3.4 Playfulness and time spent engaging in physical activity

As expected, we found positive associations between athletic identity and time spent performing PA across the total sample and among athletes and non-athletes (*r*s ≥ .26, *p*s < .001; Table 3). Hence, those with a stronger identification as an athlete reported spending more time participating in PA.

**Table 3:** Partial correlations between time spent with physical activity and the OLIW facets of playfulness and athletic identity.

Group	Other-directed	Lighthearted	Intellectual	Whimsical	Athletic identity
Total	.12* [.02, .22]	.15** [.04, .25]	.17*** [.08, .26]	.15** [.05, .25]	.52*** [.45, .59]
Non-Athletes	.06 [−.07, .19]	.03 [−.10, .17]	.02 [−.10, .15]	−.04 [−.17, .10]	.35*** [.24, .46]
Athletes	.22*** [.09, .35]	.19** [.04, .35]	.31*** [.19, .42]	.27** [.15, .39]	.26*** [.12, .38]

* *p* < .05. **p < .01. ***p < .001. Two-tailed. Bootstrapped 95% confidence intervals from 5,000 random samples in brackets. Controlled for age and gender.

All playfulness facets related positively to time spent engaged in PA, with small effect sizes (*r*s ≥ .12, *p*s ≤ .012) in the total sample. However, the fine-grained analysis of the groups showed important differences, as the correlations were negligible among non-athletes (*r*s ≤ .06), but of medium-to-large size in athletes, with *r*s between .19 (Lighthearted) and .31 (Intellectual; *p*s ≤ .005). When testing the unique contribution of the OLIW facets in a regression analysis in athletes, Intellectual (*β* = .23, *p* = .003; *Δf*^2^ = 0.10; *F*_1,201_ = 20.97; *p* < .001) and Whimsical (*β* = .16, *p* = .034 *Δf*^2^ = 0.02, *F*_1,200_ = 4.58) playfulness were predictors of time spent performing PA, explaining 11.8% of the variance, which translates to a small-to-medium regression effect size (*f*^2^ = 0.14) after controlling for age and gender (*R*^2^ = .02, *f*^2^ = 0.02).^4^

## 4 Discussion

The aim of our study was to extend the knowledge on how trait-playfulness relates to self-identification with being an athlete and time spent participating in PA. To the best of our knowledge, this is the first study to analyze the measurement invariance of playfulness among self-ascribed athletes and non-athletes. Our approach of studying athletes and non-athletes with regard to facets of playfulness and physical activity revealed both interesting and surprising findings.

We found that both groups understand the items in comparable ways and allow for comparisons between the groups, thus extending further the knowledge on the generalizability of facets of playfulness across groups (42, 43). After establishing the comparability of the playfulness measurement models between athletes and non-athletes, we found that they were similar, except for athletes showing greater Lighthearted playfulness. This is somewhat surprising given that those who view themselves as athletes and are likely to adhere to more structured trainings that require planning show more inclination to improvise in this regard than non-athletes. We supplemented self-categorization of being an athlete with a dimensional self-report measure of athletic identity (27), which mirrored the findings with participants’ self-classification by showing overall negligible to minor associations between playfulness and identifying as an athlete. An exception to this was that, among non-athletes, Other-directed playfulness yielded a small regression effect size. It might be argued that Other-directed playfulness plays a role in the perception of oneself being an athlete when it comes to team sports, as it could enable social interactions and provide a means to greater engagement in sports. This would fit well with recent findings from a diary study on the connection between playful sport design and sport engagement showing that (a) sports engagement increases when training partners are involved and that (b) the contribution of playful elements to make PA more interesting increases in a social context (17). However, the effect size is small and should be interpreted cautiously, pending replication and extension by studying its relationship with, for example, individual vs. team sports, which has been shown to relate to trait expressions of self-efficacy, positivity, resilience, self-esteem, and perseverance, to name but a few (44).

In extension to identification with being an athlete, we assessed how much time participants spent engaged in PA during their week. Again, we found playfulness was relevant among the athletes, as all types of playfulness related to spending more time with sports, yielding moderate to large correlation effect sizes. One might argue that athletes, i.e., those who engage in sports in a very structured and (semi)professional way, might use playful approaches to make it more interesting and entertaining, which then translates to more engagement with exercise and practice. Regression analyses identified Intellectual and Whimsical playfulness as relevant variables, further supporting the notion that re(framing) situations (e.g., by playing with ideas and strategies during exercise) contributes to engaging in sports. This fits well with the current literature on play and playfulness in sports. For example, Velasco and Jorda's (18) findings from various sports and activities (e.g., soccer, basketball, and triathlon) showed that boredom, especially among athletes, has detrimental consequences, including lack of participation in activities. Accordingly, (re)framing situations, especially those experienced as monotonous, might help athletes to deal with boredom and other negative consequences related to monotony and/or repetitive activities. In addition, our findings supplement those showing that the inclusion of playful elements would contribute to engaging in sports (4, 6, 17). Further research might explore person × situation interactions, testing how playful vs. less playful persons respond to PA routines that do (not) include playful elements. It would be desirable to examine the interaction between person- and situational factors that lead to engagement with sports. From the perspective of the broaden-and-build theory of positive emotions (21), it might also be argued that a playful approach to sports and the subsequent positive emotions experienced could lead to an increase in the uptake, and thereby more time spent, in participating in sports.

One might expect that the mechanisms discussed should also be found in non-athletes. Our cross-sectional data cannot provide knowledge about the causal mechanisms of playfulness and intervention studies would be needed to clarify whether changes in playfulness would accompany changes in athletes’ subjective outcomes, such as boredom during training or objective indicators such as time spent with PA [see also (4) and (5)]. One avenue to learning more about the role of playfulness among non-athletes might be focusing on the relation to Other-directed playfulness. As demonstrated in the field of relationships (9), playfulness provides means to establish and maintain social connections, and this in turn could contribute to people, especially non-athletes, engaging in sports as a “by-product,” especially when social connections might be made and maintained for example, by being a member in a sports group [e.g., (45–47)].

In short, playfulness may serve as an important psychological resource in the context of training, be it in leisure-time or (semi-)professionally, particularly when exercises become repetitive, monotonous, or mentally taxing, and when additional motivation is needed. Engaging in regular training routines often requires sustained motivation, especially when progress is slow, tasks lack variety, or when training is undertaken alone with little distraction and/or under challenging or harsh conditions. In such situations, boredom can become a barrier to adherence. Adult playfulness, particularly in its lighthearted and other-directed forms, may help individuals reframe repetitive exercises in a more enjoyable and engaging manner. For example, playful individuals might infuse their routines with small challenges, unconventional ideas and thoughts, or small games (e.g., in social settings), thereby increasing the perceived fun of the activity—and interest and engagement with the activity itself. This reframing may not only reduce boredom but may also foster positive affect and intrinsic motivation, both of which are crucial for long-term commitment to physical activity. Playfulness may function as a buffer against the demotivating and difficult effects of routine, enabling individuals to persist in training programs and potentially achieve better outcomes in physical fitness and well-being.

### 4.1 Limitations and future directions

Our study has several limitations. First, we relied on self-reports for all study variables. While there is robust evidence that self-reports of playfulness and time spent participating in PA are comparatively accurate [e.g., (32, 48)], supplementing the self-reports with objective recordings and reports by knowledgeable others [see (26)] is desirable in future studies to reduce method variance that can inflate correlations. Further, an objective and valid criterion for assessing the status of being an athlete or non-athlete is hitherto difficult to establish [see also (30)]. Classification is usually based on several factors such as length of athletic career, participation in competitions, gender, and age (28), but there is no consensus on the definition of athlete status. Although the WHO specifies the length of time that distinguishes between what is considered not enough and what is healthy exercise (49), this alone is not sufficient to determine athlete status and, accordingly, we relied on the frequently used method of self-identification. Also, it is unclear whether or to what degree self-selection plays a role for the findings. While we contacted sports clubs and invited members to participate, it is unclear how well our subgroups represent the underlying populations of athletes and non-athletes. Secondly, our findings are limited to German-speakers, and we have not tested professional athletes. Thirdly, we have not distinguished between the type of sports that our participants engage in. As discussed, the type of sports undertaken might relate to playfulness, for example: whether one engages in a play-based sports with rules (e.g., soccer or ice hockey) vs. activities in which there is an absence of formal rules (e.g., free swimming); team- vs. individual sports; and sports that relate to monotony [e.g., swimming the same 25 m track in a pool 100 times on a daily basis vs. being able to engage with a new environment daily, such as in long-distance running; (18)]. Fourth, women are overrepresented in our sample of non-athletes. Although the consideration of gender as covariate did not point to robust effects, replication using more balanced samples is desirable to ensure the distinction between effects of group and gender. Finally, our data are cross-sectional and do not allow conclusions about the mechanisms behind our findings. While playfulness is a personality trait that is stable across time and situations, it is feasible that engagement in sports in adolescence might affect playfulness and attitudes toward sports and PA during adolescence [e.g., (50)]. Also, people differ regarding their motives for participating in PA, and it is feasible that personal motives (e.g., enjoyment vs. others’ expectations) affect how people experience and approach PA and how playfulness contributes to meeting one's activity-related goals (51).

We hope that our findings supplement and stimulate research on the role of play and playfulness in sports as a potential facilitator of engaging in PA. There are fruitful research avenues from the growing literature on play(fulness) in sports. Beyond studying person × situation interactions, trainings of trait-playfulness might be tested in future research. In a randomized placebo-controlled study, Proyer et al. (52) showed that playfulness can be stimulated and that changes in playfulness go along with greater well-being and reduced depressiveness. We recommend that future research should examine whether interventions might provide insights into the mechanisms and might translate from athletes to non-athletes in terms of time spent participating in PA, as it might be feasible that greater playfulness also supports non-athletes in engaging in being active. Further, interventions might be adapted to the context of sports by asking participants to actively incorporate playful elements into their PA. We would expect that this might contribute to an increase in the joy of engaging in PA (4).

## Data Availability

The datasets presented in this study can be found in online repositories. The names of the repository/repositories and accession number(s) can be found below: all data and syntaxes are openly available in the Open Science Framework (https://osf.io/gpbe9/).

## Footnotes

^1^We use the term “physical activity” to mean sports-related physical activity involving bodily movement that is perceived as sports, irrespective of whether it follows formal rules.

^2^The low number of items and the breadth of content covered in the scale yields typically comparatively low internal consistency values. Alternative approaches to estimating reliability support the use of the scale. For example, Proyer (7) found robust temporal stability, with test-retest correlations of .73 (1-week), .74 (2-week and 1-month), and .67 (3-month interval), and inter-rater reliability at zero-acquaintance is .69 and .72 [(33, 34)].

^3^All variance inflation factors were ≤ 1.66 and tolerance values ≥ 0.6, thus, not indicating multicollinearity.

^4^When entering all OLIW facets as predictors, the explained variance does not change.
